# Effects of Dietary Carbohydrate Levels on Growth and Ammonia Excretion in Chinese Perch (*Siniperca chuatsi*) at Low Water Temperatures

**DOI:** 10.3390/ijms26104638

**Published:** 2025-05-13

**Authors:** Yufei Zhang, Lingchen Fang, Zhiwei Zou, Jianmei Su, Liwei Liu

**Affiliations:** 1 College of Fisheries, Chinese Perch Research Center, Engineering Research Center of Green Development for Conventional Aquatic Biological Industry in the Yangtze River Economic Belt, Ministry of Education, Huazhong Agricultural University, Wuhan 430070, China; zyufei@webmail.hzau.edu.cn (Y.Z.); 2024308120052@webmail.hzau.edu.cn (L.F.); 2 Hubei Key Laboratory of Regional Development and Environmental Response, Faculty of Resources and Environmental Science, Hubei University, Wuhan 430062, China; 202421108012111@stu.hubu.edu.cn

**Keywords:** deamination, glucose metabolism, high-carbohydrate

## Abstract

This study investigated the effects of dietary carbohydrate levels (control 8.13%, HG1 12.03%, and HG2 14.15%) on growth performance and glutamate metabolism in Chinese perch (*S. chuatsi*) (initial weight: 39.12 ± 0.25 g) reared at 12–15 °C. Diets were isonitrogenous (49% protein). After 8 weeks, the HG1 group optimized weight gain rate (WGR), specific growth rate (SGR), and protein efficiency ratio (PER), while reducing feed conversion ratio (FCR). HG1 and HG2 groups reduced liver glutamate/glutamine levels while downregulating the expression of key ammonia-metabolizing genes (*gs*, *gdh*, and *ampd*), collectively suppressing glutamate-mediated ammonia excretion. HG1 and HG2 groups enhanced glycolysis (upregulated *gk* and *pk*) coupled with suppressed gluconeogenesis (decreased PEPCK and G6Pase activities) in the liver. Concurrent downregulation of proteolytic markers (*mafbx* and *murf1*) in the muscle indicated improved protein conservation efficiency in the HG1 and HG2 groups. The HG1 diet optimally enhances growth by promoting glycolysis, reducing ammonia excretion, and improving feed efficiency. The insights gained from this research will be used to refine the low-temperature culture feed for Chinese perch, aiming to decrease ammonia and nitrogen emissions, thereby advancing the practice of low-ammonia emission culture for this species.

## 1. Introduction

Carbohydrates represent the most economical energy source in compound feeds. Incorporating optimal carbohydrate levels in basal diets can spare proteins from being used as energy substrates, thereby promoting fish growth while enhancing antioxidant capacity and immune function [1]. Unlike proteins, carbohydrates do not generate ammonia nitrogen during metabolism, thus mitigating aquaculture water pollution. However, fish —particularly carnivorous species—possess a limited capacity for dietary carbohydrate utilization. High-carbohydrate diets may lead to persistent hyperglycemia, metabolic disorders, growth suppression, and reduced feed efficiency [2]. Furthermore, excessive dietary carbohydrate intake promotes liver lipid accumulation in fish, significantly elevating whole-fish crude lipid content [3]. This metabolic disturbance subsequently induces pathological alterations, including hepatomegaly and cytoplasmic vacuolization, ultimately compromising liver function [4]. Therefore, the scientific optimization of dietary carbohydrate levels is essential. Appropriate carbohydrate regulation not only enhances fish growth performance but also reduces production costs and improves farming efficiency. The metabolism of carbohydrates by fish is influenced by multiple factors, including carbohydrate type, dietary inclusion level, feeding ecology, and environmental conditions [5,6].

Studies in common carp (*Cyprinus carpio*) have demonstrated that high-carbohydrate diets not only promote protein sparing for energy utilization but also significantly decrease nitrogenous waste excretion through modulation of proteolytic enzyme activities [7]. Although fish primarily rely on amino acid oxidation from protein hydrolysis for energy production and exhibit limited carbohydrate utilization capacity, high-carbohydrate diets may alter this metabolic pathway. In teleost fish, ammonia primarily originates from amino acid catabolism, and its accumulation can be toxic [8]. Within energy metabolism, amino acid deamidation plays a vital role, with glutamate (Glu) and glutamine (Gln) serving as primary energy substrates [9]. Through glutamate dehydrogenase (GDH) catalysis, glutamate is converted to α-ketoglutarate (AKG), which subsequently enters the tricarboxylic acid (TCA) cycle, generating energy via oxidative deamination [10].

As poikilothermic animals, fish experience pronounced temperature-dependent modulation of growth and developmental processes [11]. Within their optimal thermal range, fish achieve physiological adaptation to temperature fluctuations through metabolic regulation mechanisms [12]. However, when the water temperature exceeds the tolerance threshold of fish, it may disrupt their physiological and biochemical functions, causing tissue damage and even death [13]. Under low temperatures (3.6–18 °C), fish are susceptible to starvation, which can lead to a decrease in body mass and organ indices, which in turn leads to overwintering losses of cultured fish [14,15]. Although numerous studies have examined carbohydrate level effects on growth performance in grass carp (*Ctenopharyngodon idella*) [16], yellow catfish (*Pelteobagrus fulvidraco*) [17], turbot (*Scophthalmus maximus*) [18], and groupers (*Epinephelus*) [19], relatively few studies have been conducted on the effects of carbohydrate levels on growth, amino acid metabolism, and carbohydrate metabolism of fish under chronic hypothermic conditions. Therefore, an in-depth exploration of this area is important for optimizing fish culture management strategies, green farming, and improving culture efficiency.

Chinese perch (*S. chuatsi*) represents a commercially important freshwater carnivorous species, valued for its absence of intermuscular spines and notable nutritional and medicinal benefits [20,21]. Following successful domestication, this species has achieved consistent acceptance of formulated feeds, enabling sustainable intensive aquaculture. Although Chinese perch maintained feeding activity during winter, ammonia excretion rates remained significantly elevated in fish fed diets. However, limited information is available regarding how dietary carbohydrate levels affect glutamate metabolism and ammonia excretion in Chinese perch under low-temperature winter conditions. This study systematically investigates the effects of graded dietary carbohydrate levels on growth performance, glutamate metabolism, and carbohydrate utilization in Chinese perch. The findings aim to provide a scientific basis for developing environmentally sustainable, low-ammonia emission feeds optimized for low-temperature aquaculture conditions.

## 2. Results

### 2.1. Effect of Dietary Carbohydrate Levels on the Growth Performance Under Low Water Temperature

The initial body weight (IBW) of carbohydrate groups is shown in Table 1. Different dietary carbohydrate levels had no significant effect on the survival rate (SR) or feeding rate (FR) of Chinese perch. The final mean body weight (FBW), weight gain rate (WGR), specific growth rate (SGR), protein retention rate (PR), and protein efficiency ratio (PER) of Chinese perch showed a trend of initially increasing and then decreasing with increasing dietary carbohydrate levels. These parameters were significantly higher (*p* < 0.05) in the HG1 group, while no significant differences were observed between the HG2 group and the control group. Additionally, the feed conversion ratio (FCR) was significantly lower (*p* < 0.05) in the HG1 group, with no significant difference between the HG2 and control groups.

### 2.2. Effects of Dietary Carbohydrate Levels on Morphometric Indices and Body Composition Under Low Water Temperature

According to Table 2, dietary carbohydrate levels significantly affected the morphometric indices of Chinese perch. Although no significant effect was observed on the hepatosomatic index (HSI), the viscerosomatic index (VSI) increased with increasing dietary carbohydrate levels. The HG2 group showed the highest VSI (*p* < 0.05), with no significant difference between the HG1 and control groups. Conversely, the condition factor (CF) of Chinese perch in the HG2 group was significantly lower than in both the HG1 and control groups (*p* < 0.05).

As shown in Table 3, dietary carbohydrate levels had no significant effect on body composition parameters, including crude fat content, crude protein content, ash content, or moisture content of Chinese perch.

### 2.3. Effects of Dietary Carbohydrate Levels on Glutamate Metabolism Under Low Water Temperature

**Figure 1:**
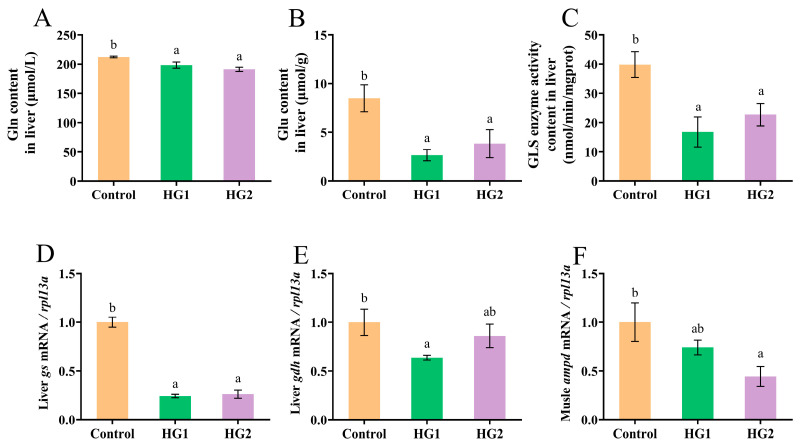
Effects of different dietary carbohydrate levels on glutamate metabolism of Chinese perch at low water temperature. (**A**) Gln content; (**B**) Glu content; (**C**) GLS enzyme activity content; (**D**) Relative expression of *gs*; (**E**) Relative expression of *gdh*; (**F**) Relative expression of *ampd*. The data were shown as mean ± standard error (*n *= 9), with different letters indicating significant differences among different groups (*p* < 0.05); the same applies below.

As shown in Figure 1, increasing dietary carbohydrate levels significantly reduced liver glutamine (Gln) content, glutamate (Glu) content, glutaminase (GLS) activity, and the relative expression of the *gs* gene in Chinese perch (Figure 1A–D). All experimental groups exhibited significantly lower values for these parameters compared to the control group (*p* < 0.05), with no significant differences observed between the HG1 and HG2 groups. Additionally, both experimental groups showed reduced relative expression of the liver *gdh* gene and the muscular *ampd* gene compared to those in the control group. Specifically, the HG1 group demonstrated significantly lower liver *gdh* expression (Figure 1E), while the HG2 group showed significantly lower muscular *ampd* expression (Figure 1F) (*p* < 0.05).

### 2.4. Effect of Dietary Carbohydrate Levels on Ammonia Excretion Under Low Water Temperature

**Figure 2:**
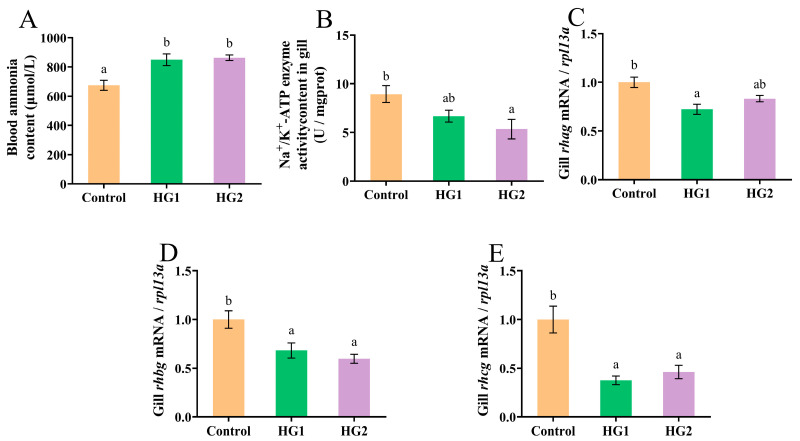
Effects of different dietary carbohydrate levels on ammonia excretion of Chinese perch at low water temperature. (**A**) Blood ammonia content; (**B**) Na^+^/K^+^-ATPase activity content; (**C**) Relative expression of *rhag*; (**D**) Relative expression of *rhbg*; (**E**) Relative expression of *rhcg*. The data were shown as mean ± standard error (*n *= 9), with different letters indicating significant differences among different groups (*p* < 0.05).

The influence of dietary carbohydrate levels on ammonia excretion in Chinese perch is presented in Figure 2. Blood ammonia concentrations (Figure 2A) were significantly elevated (*p* < 0.05) in all experimental groups relative to the control, with no significant difference observed between the HG1 and HG2 groups. The activity of the ammonia-nitrogen transporter enzyme Na^+^/K^+^-ATPase in gill filaments (Figure 2B) exhibited a dose-dependent decrease with increasing dietary carbohydrate levels. Specifically, Na^+^/K^+^-ATPase activity was significantly lower (*p* < 0.05) in the HG2 group compared to the control group. Dietary carbohydrate levels significantly modulated the expression of ammonia transporter genes (*rhag*, *rhbg*, and *rhcg*) in gill filaments. The relative expression of *rhag* (Figure 2C) was significantly downregulated (*p* < 0.05) in the HG1 group compared to the control group. Conversely, the control group showed significant upregulation (*p* < 0.05) of both *rhbg* and *rhcg* genes (Figure 2D,E).

### 2.5. Effects of Dietary Carbohydrate Levels on Glycolipid Metabolism Under Low Water Temperature

**Figure 3:**
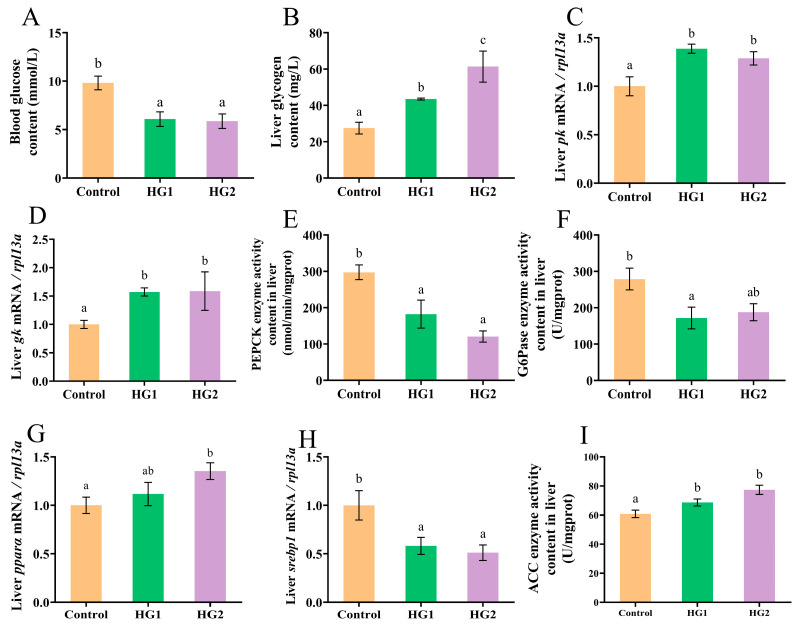
Effects of different dietary carbohydrate levels on glucose and lipid metabolism of Chinese perch at low water temperature. (**A**) Blood glucose content; (**B**) Liver glucose content; (**C**) Relative expression of *pk*; (**D**) Relative expression of *gk*; (**E**) PEPCK enzyme activity content; (**F**) G6Pase enzyme activity content; (**G**) Relative expression of *ppara*; (**H**) Relative expression of *srebp1*; (**I**) ACC enzyme activity content. The data were shown as mean ± standard error (*n *= 9), with different letters indicating significant differences among different groups (*p* < 0.05).

The effects of dietary carbohydrate levels on the glycolipid metabolism in Chinese perch are presented in Figure 3. Blood glucose levels (Figure 3A) were significantly decreased (*p* < 0.05) in both the HG1 and HG2 groups compared to the control. Liver glycogen contents (Figure 3B) showed a dose-dependent increase, with the HG2 group being significantly higher than both the HG1 and control groups (*p* < 0.05), and the HG1 group significantly exceeding the control.

The relative expression of key glycolytic genes (*pk* and *gk*) in the liver (Figure 3C,D) was significantly upregulated (*p* < 0.05) in both HG1 and HG2 groups compared to the control, indicating enhanced liver glycolysis with increasing dietary carbohydrates. Conversely, activities of gluconeogenic enzymes PEPCK and G6Pase (Figure 3E,F) were significantly reduced (*p* < 0.05) in the HG1 group compared to the control, suggesting carbohydrate-mediated suppression of gluconeogenesis.

Lipid metabolism markers showed distinct responses: *pparα* gene expression was significantly downregulated (*p* < 0.05) in the control versus the HG2 group (Figure 3G), while *srebp1* expression was significantly upregulated (Figure 3H). Acetyl-CoA carboxylase (ACC) enzyme activities (Figure 3I) were significantly increased (*p* < 0.05) in both HG1 and HG2 groups compared to the control.

### 2.6. Effects of Dietary Carbohydrate Levels on the AMPK Signaling Pathway Under Low Water Temperature

**Figure 4:**
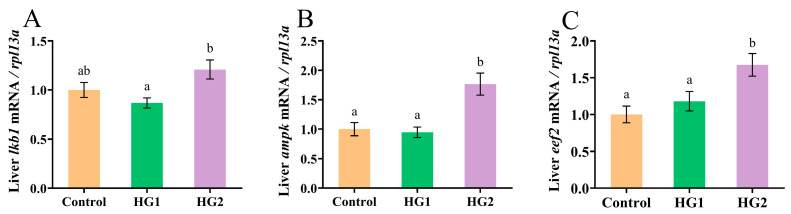
Effects of different dietary carbohydrate levels on the AMPK signaling pathway in Chinese perch at low water temperature. (**A**) Relative expression of *lkb1*; (**B**) Relative expression of *ampk*; (**C**) Relative expression of *eef2*. The data were shown as mean ± standard error (*n* = 9), with different letters indicating significant differences among different groups (*p* < 0.05).

Figure 4 shows the effects of dietary carbohydrate levels on AMPK signaling pathway-related gene expression in Chinese perch. The relative expression of liver *lkb1* and *ampk* exhibited a biphasic response, initially decreasing and then increasing with elevated dietary carbohydrates, reaching significantly the highest levels (*p* < 0.05) in the HG2 group. Furthermore, the expression of the *eef2* demonstrated a dose-dependent increase, with the HG2 group showing significantly higher expression (*p* < 0.05) compared to both the HG1 and control groups.

### 2.7. Effect of Dietary Carbohydrate Levels on Protein Synthesis and Catabolism Under Low Water Temperature

**Figure 5:**
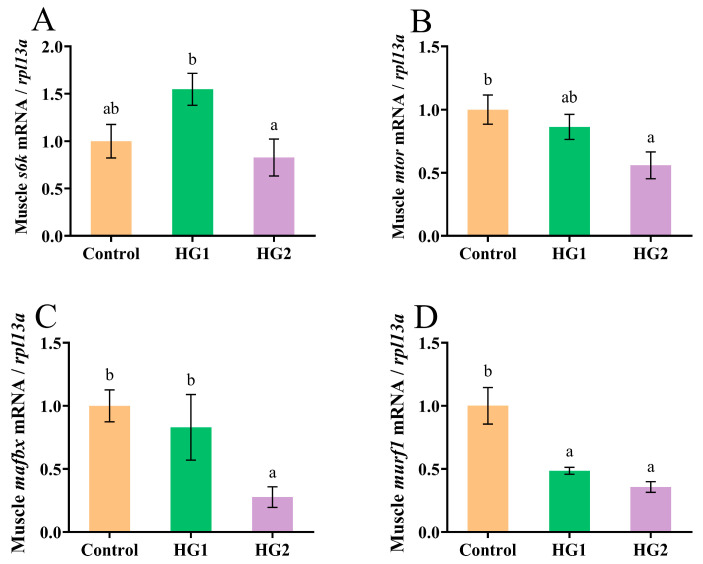
Effects of different dietary carbohydrate levels on protein synthesis and decomposition of Chinese perch at low water temperature. (**A**) Relative expression of *s6k1*; (**B**) Relative expression of *mtor*; (**C**) Relative expression of *mafbx*; (**D**). Relative expression of *murf1*. The data were shown as mean ± standard error (*n *= 9), with different letters indicating significant differences among different groups (*p* < 0.05).

Figure 5 shows the significant alterations in protein metabolism-related gene expression in Chinese perch muscle under the different diets. The relative expression of mTOR signaling pathway regulators (*s6k1* and *mtor*) is shown in Figure 5A,B. Notably, *s6k1* expression was significantly upregulated in the HG1 group compared to the HG2 group (*p* < 0.05), while *mtor* expression was significantly downregulated in the HG2 group in comparison to the control (*p* < 0.05). Figure 5C,D show the expression patterns of proteolysis-related genes (*mafbx* and *murf1*). The HG2 group exhibited significant downregulation of *mafbx* expression compared to the control (*p* < 0.05). Furthermore, both the HG1 and HG2 groups showed significantly reduced *murf1* expression (*p* < 0.05), with the lowest levels observed in the HG2 group.

## 3. Discussion

Carbohydrate represents a highly abundant and economically viable nutrient source in aquaculture [22]. Although moderate dietary carbohydrate supplementation has been shown to enhance fish growth performance [23,24], long-term high carbohydrate intake may have inhibitory effects on growth [25,26]. This phenomenon has been well-documented across multiple species. In yellow catfish (*Pelteobagrus fulvidraco*), dietary carbohydrate levels of 31.11% significantly reduced WGR and SGR compared to control diets containing 23.25% carbohydrates [17]. Similarly, Xing et al. [27] reported that while a carbohydrate level of 26.69% improved growth performance in yellow catfish, levels exceeding this threshold resulted in decreased SGR. Consistent with these findings, our experimental results revealed that Chinese perch fed a 14.15% (HG2) carbohydrate diet exhibited significantly lower WGR and SGR compared to those receiving a 12.03% (HG1) carbohydrate formulation. This pattern of growth reduction beyond optimal carbohydrate levels has been consistently observed in diverse species, including Nile tilapia (*Oreochromis niloticus*) [28], orange-spotted grouper (*Epinephelus coioides*) [29], and crucian carp (*Carassius cuvieri*) [30], where dietary carbohydrate levels exceeding the optimal range consistently reduced WGR and SGR. Another study further established that Chinese perch (*S. chuatsi*) shows optimal growth performance with dietary starch inclusion levels ranging from 8% to 10%, significantly outperforming both higher and lower carbohydrate formulations [31]. These findings align with the results of the current study, in which Chinese perch exhibited significantly the highest WGR, SGR, PER, and PR at a feed starch addition of 10% and a carbohydrate level of 12.03%. This optimal growth performance may result from enhanced protein synthesis and metabolic efficiency facilitated by appropriate carbohydrate inclusion, thereby improving overall nutrient absorption and utilization.

The liver serves as both a nutrient storage organ and the primary site of energy metabolism in fish. Morphological indices, including CF, HSI, and VSI, are widely used to evaluate growth and developmental status [32]. During normal growth progression, decreasing HSI and VSI values typically reflect enhanced metabolic efficiency in nutrient conversion and utilization to support developmental energy requirements [33]. High-carbohydrate diets are known to stimulate liver glycogen deposition through enhanced glycogenesis, often resulting in significantly elevated HSI values [34,35]. In this experiment, Chinese perch displayed dose-dependent increases in VSI with rising dietary carbohydrate levels, peaking in the HG2 group. Although the high-carbohydrate groups showed elevated HSI values compared to the control, the differences were not statistically significant. These morphological alterations likely result from carbohydrate-induced lipid deposition in the liver and visceral tissues, ultimately increasing visceral mass. This could be attributed to the fact that the increase in carbohydrate level led to the accumulation of fat in the liver of Chinese perch, which in turn increased the weight of the visceral mass. This phenomenon has been similarly documented in other fish species, including crucian carp (*C. cuvieri*) and large yellow croaker (*Larimichthys crocea*) [27,30]. Notably, CF was significantly lower in the HG2 group, indicating potential metabolic dysregulation in lipid-carbohydrate homeostasis at excessive dietary carbohydrate levels [36]. This finding contrasts with studies on juvenile starry flounder (*Platichthys stellatus*), showing no significant growth impact from dietary carbohydrate variation [37]. This discrepancy may be attributed to factors such as differences in the experimental fish species and in dietary habits, diversity of feed carbohydrate sources, changes in the culture environment, and the length of the experimental period.

In fish metabolism, Glu and Gln are the main carriers of nitrogen. Gln is converted to Glu through GLS activity, with Glu functioning as both a key intermediate in the TCA cycle and a substrate for Gln regeneration via GS [38]. This metabolic cycling between Glu and Gln plays an essential role in nitrogen storage, transport, and utilization [39]. Furthermore, the process generates AKG, a crucial metabolite that participates in cellular energy production [40]. Additionally, the GS/GLS-mediated interconversion system is vital for maintaining whole-body ammonia homeostasis, particularly during periods of metabolic stress or altered nitrogen balance [10,38]. Fish primarily excrete ammonia via two mechanisms: (i) the ornithine-urea cycle (resulting in urea production); and (ii) the glutamine metabolic pathway via gill tissues [41]. In the present study, GLS enzyme activity, relative expression of the *gs* gene, and Glu and Gln contents were significantly reduced in the HG1 and HG2 groups compared to the control, demonstrating suppression of the glutamine-mediated excretion pathway under high dietary carbohydrate conditions. These findings are consistent with observations in Nile tilapia (*Oreochromis niloticus*), where inhibition of glutamate metabolism similarly reduced Glu and Gln levels [42], further supporting the predominant role of the glutamine pathway in piscine ammonia excretion. In the current study, elevated blood ammonia levels were observed in the HG1 and HG2 groups, mirroring findings in Chinese mitten crab (*Eriocheir sinensis*), where reduced ammonia excretion led to blood ammonia accumulation [43]. GDH catalyzes the reversible conversion between Glu and AKG, playing a pivotal role in ammonia metabolism regulation, with *gdh* and *ampd* genes serving as crucial deamination regulators in fish [44,45]. The results showed downregulated expression of these genes in Chinese perch under high-glucose conditions, indicating impaired liver deamination capacity that consequently affects ammonia excretion. That is, reduced GDH enzyme activity decreases the liver ammonia deamination in fish [46].

The gill represents a critical target organ in fish ammonia metabolism, with its basolateral membrane containing the Na^+^/K^+^-ATPase, and this enzyme plays a key role in ammonia transport [47]. The results of the present study demonstrated significantly reduced Na^+^/K^+^-ATPase activity in the HG2 group, suggesting impaired ammonia-nitrogen metabolism in Chinese perch. This enzymatic activity may result from structural modifications of the Na^+^/K^+^-ATPase protein complex [48]. In addition, the Rhesus (Rh) glycoprotein family in gill tissues, comprising Rhag, Rhbg, and Rhcg, plays a vital role in ammonia transmembrane transport. These proteins are essential for regulating blood and tissue ammonia concentrations, thereby maintaining nitrogen balance [49]. In this experiment, the relative expression of gill *rhag*, *rhbg*, and *rhcg* genes was significantly downregulated in the high-carbohydrate groups. These results were consistent with a previous study, which suggested that compromised ammonia transport efficiency may reduce excretory capacity [50].

Glycolysis and gluconeogenesis represent the two fundamental pathways of carbohydrate metabolism, governing carbohydrate catabolism and synthesis, respectively. In glycolysis, glucokinase (GK) and pyruvate kinase (PK) serve as the primary rate-limiting enzymes [51], whereas phosphoenolpyruvate carboxykinase (PEPCK) and glucose-6-phosphatase (G6Pase) fulfill this role in gluconeogenesis [52]. Previous studies have established that liver *gk* expression in carnivorous fish is predominantly regulated by dietary carbohydrate levels [53]. In the present study, the relative expression of *pk* and *gk* genes was upregulated in the HG1 and HG2 groups, while the enzyme activities of PEPCK and G6Pase were decreased. These findings suggest that elevated dietary carbohydrates enhanced liver glycolysis while suppressing gluconeogenesis in Chinese perch under low-temperature conditions. This metabolic shift likely contributed to improved glycemic control, as evidenced by reduced blood glucose levels in high-carbohydrate-fed fish. Consistent with these findings, high-carbohydrate diets were shown to significantly enhance the liver PK activity in large yellow croaker (*L. crocea*) [27] and perch (*Perca fluviatilis*) [54], while suppressing *pepck* expression in turbot (*S. maximus*) [55]. In addition, in this experiment, the high-carbohydrate group (HG1 and HG2) exhibited reduced blood glucose levels alongside increased liver glycogen content. These results demonstrate that elevated dietary carbohydrates promote glucose accumulation in fish liver, leading to significantly enhanced glycogen synthesis and storage. This metabolic shift effectively reduces circulating glucose levels through liver sequestration.

Sterol regulatory element-binding protein 1 (SREBP1) serves as a master transcriptional regulator of lipogenesis, while peroxisome proliferator-activated receptor α (PPARα) functions as a nuclear receptor promoting fatty acid β-oxidation and energy metabolism [56]. In this study, the expression of *pparα* gene was significantly upregulated in the HG2 group, showing a positive correlation with dietary carbohydrate levels. Conversely, *srebp1* gene expression was downregulated, indicating suppressed fatty acid and cholesterol synthesis alongside enhanced fatty acid oxidation to meet cellular energy demands. Interestingly, despite the overall promotion of fatty acid oxidation, elevated acetyl-CoA carboxylase (ACC) activity in high-glucose groups suggested concurrent stimulation of certain lipogenic pathways. AMP-activated protein kinase (AMPK), a central cellular energy sensor, critically regulates liver glucose and lipid metabolism [57]. Notably, the control group exhibited downregulated expression of genes associated with the AMPK signaling pathway, which may disrupt metabolic homeostasis. In the HG2 group of this experiment, the expression of AMPK signaling pathway-related genes (*lkb1*, *ampk*, and *eef2*) was upregulated. This AMPK activation likely enhanced fatty acid oxidation, increasing cellular acetyl-CoA concentrations—a potential mechanism underlying the observed elevation in ACC activity. Furthermore, AMPK-mediated suppression of liver gluconeogenesis was evidenced by reduced PEPCK and G6Pase activities, a result which was consistent with our experimental results. This metabolic shift contributed to improved glycemic control through decreased fasting blood glucose levels.

Dietary carbohydrate supplementation at appropriate levels significantly enhances protein utilization efficiency. In the present study, the HG1 group demonstrated the highest PER and PR, indicating that a 12.03% carbohydrate level maximizes protein utilization in Chinese perch under low-temperature conditions. This protein-sparing effect likely results from glucose metabolism inhibiting key gluconeogenic enzymes (PEPCK and G6Pase), thereby reducing amino acid catabolism for energy production. These metabolic adjustments promote the allocation of dietary protein toward growth rather than energy metabolism [27]. This is consistent with the results of this experiment, which show that gluconeogenesis was inhibited in the high-carbohydrate group. The mTOR and AMPK signaling pathways play key roles in regulating protein utilization and energy metabolism [58]. In the present study, the AMPK signaling pathway was activated in the HG2 group, indicating enhanced catabolic activity in Chinese perch fed high-carbohydrate diets, concurrent with inhibition of protein synthesis pathways. This metabolic profile corresponds with the lowest PER observed in the HG2 group. Notably, the HG1 group exhibited upregulated expression of the mTOR-related *s6k1* gene, potentially enhancing protein synthesis under low-temperature conditions. Furthermore, reduced expression of proteolysis-related genes (*murf1* and *mafbx*) in high-glucose groups suggests decreased muscle protein degradation, consistent with the optimal protein efficiency ratio and protein retention observed in the HG1 group. Concurrently, the HG1 group exhibited significant upregulation of key glycolytic genes (*gk* and *pk*), enhancing carbohydrate digestion and absorption. This metabolic shift reduced dependence on protein catabolism for energy, thereby improving feed utilization efficiency and promoting protein conservation.

## 4. Materials and Methods

### 4.1. Fish Maintenance

The experimental fish were selected from the Chinese perch Breeding Innovation Base of Huazhong Agricultural University. After 4 weeks of preliminary rearing on palatable diets, 270 Chinese perch (initial body weight: 39.12 ± 0.25 g) from the same cohort, free of injuries and exhibiting normal feeding behavior, were selected. The fish were randomly assigned to 9 recirculating aquaculture tanks (water volume: 1.75 m^3^) and cultured for 8 weeks, with 30 individuals per tank, resulting in a stocking density of 17 fish/m^3^. Each experimental group is equipped with three parallel aquaculture tanks (*n* = 3 replicates per group). Dissolved oxygen concentration in the culture water was controlled at 7–8 mg/L, temperature at 12–15 °C, and pH at 7.8–8.1. Feeding was conducted at fixed times (10:00 and 17:00) every day. After feeding, the feces were removed at 14:00, and the water was replaced. Due to the influence of feeding in winter, 1% of the initial body weight was used for quantitative feeding throughout the experiment. The food intake of each tank was recorded and counted every day.

### 4.2. Experimental Diets

The test feeds were formulated using fish meal, fermented soybean meal, chicken meal, and gelatin as protein sources; ish oil and soybean oil as fat sources; and cornstarch as a carbohydrate source. Three isonitrogenous and isoenergetic diets with graded carbohydrate levels (control 8.13%, HG1 12.03%, and HG2 14.15%) were prepared. The detailed nutrient compositions are presented in Table 4. All the dry matter ingredients were carefully ground through a 60-mesh sieve, stirred and mixed gradually in the order of their proportions, and the trace amounts of premix were mixed by gradual dilution. The prepared feeds were put into self-sealing bags according to the experimental groups and stored at −20 °C.

### 4.3. Sample Collection

At the end of the culture experiment, the experimental fish were fasted for 24 h. The culture water was lowered, and the fish were anesthetized with MS-222 (150 mg/L) until they showed no obvious movement, then harvested and weighed. In each tank, nine Chinese perch were randomly selected, and venous blood samples were collected. The blood was centrifuged at 3000 rpm for 15 min at 4 °C, then stored for 1–2 h before collecting the supernatant for blood glucose determination. Then the viscera and liver were dissected and weighed to calculate the viscera-body and liver-body ratios. The gills, liver, and back muscles were sequentially clipped and placed in 2 mL enzyme-free EP tubes, then stored at −80 °C for further analysis.

### 4.4. Biochemical Parameters Analysis

Blood glucose, blood ammonia, liver glycogen content, glutaminase (GLS) activity, and Na^+^/K^+^-ATPase activity were measured using commercial assay kits (Item No. A154-1-1, A086-1-1, A043-1-1, A124-1-1, A070-2-2; Nanjing Jianjian Bioengineering Research Institute, Nanjing, China). Glutamic acid (Glu) content was determined using the kit (Item No. BC1580; Beijing Solepol Technology Co., Beijing, China). Phosphoenolpyruvate carboxykinase (PEPCK) activity, glucose-6-phosphatase (G6Pase) activity, and acetyl coenzyme A carboxylase (ACC) activity were determined by using the kits (Item No. YX-C-C500, LE-2-350, LE-2-148; Hefei Lair Biotechnology Co. Ltd., Hefei, China).

Primers were designed based on Chinese perch gene sequences from GenBank (https://www.ncbi.nlm.nih.gov/genbank/, accessed on 6 April 2025) (Table 5) and synthesized by Sangong Bioengineering Co (Shanghai, China). Total RNA was extracted from tissues using TRIzol reagent (Invitrogen, Carlsbad, CA, USA). cDNA was synthesized using the Evo M-MLV Reverse Transcription Premixed Kit (Accurate Biology, Changsha, China) and stored at −20 °C. RT-qPCR was performed on a LightCycler 480 II system (Roche, Berlin, Germany). The housekeeping gene *rpl13a* was used for normalization, and gene expression levels were calculated using the 2^−ΔΔCt^ method according to the protocol of Su et al. [59,60].

### 4.5. Calculation and Statistical Analysis

The following growth parameters were calculated:

Survival rate (SR %) = (final fish number/initial fish number) × 100.

Weight gain rate (WGR %) = (final body weight − initial body weight)/initial body weight × 100

Specific growth rate (SGR %/day) = (Ln final body weight − Ln initial body weight)/days × 100.

Feeding rate (FR %/day) = total feed intake/[(final body weight − initial body weight)/2]/days × 100.

Feed conversion ratio (FCR) = feed intake/(final weight − initial weight).

Protein efficiency ratio (PER) = (final body weight − initial body weight)/protein intake.

Protein retention efficiency (PR %) = 100 × protein gain/protein intake.

Hepatosomatic index (HSI %) = 100 × (liver weight/final body weight).

Viscerosomatic index (VSI %) = 100 × (visceral weight/final body weight).

Condition factor (CF, g/cm^3^) = 100 × (W/L^3^), (W: body weight; L: standard length)

All data in the experiment are expressed as mean ± standard error (mean ± S.E.). Statistical analyses were performed using SPSS 25.0 software, and one sample *t*-test was performed on the same data set to exclude samples with large deviations from the overall mean. A one-way ANOVA was applied to compare growth and body composition data between groups, followed by a Duncan test with a significant difference of *p *< 0.05.

## 5. Conclusions

Under low-temperature culture conditions, Chinese perch exhibit reduced feed intake. This study demonstrates that increasing dietary carbohydrate levels to 12.03% (HG1 group) significantly enhanced WGR and SGR. This nutritional intervention simultaneously upregulated liver expression of glycolytic key enzymes (*gk* and *pk*) while suppressing gluconeogenic enzyme activities (PEPCK and G6Pase). The metabolic shift activated the AMPK signaling pathway, coordinating glucose-lipid metabolism to enhance non-protein energy supply while reducing dependence on protein catabolism. Consequently, PER and PR were significantly improved. The high-carbohydrate diet further modulated nitrogen metabolism by inhibiting the glutamine metabolic pathway, downregulating the liver *gdh* expression, and impairing gill ammonia transport through reduced Na^+^/K^+^-ATPase activity. These metabolic adaptations collectively reduced ammonia excretion while promoting protein anabolism in Chinese perch. The study provides crucial insights for developing low-ammonia emission culture systems under low-temperature conditions, advancing environmentally sustainable Chinese perch aquaculture practices.

## Figures and Tables

**Table 1:** Effects of dietary carbohydrate levels on growth performance of Chinese perch at low water temperature (*n* = 9).

Parameters	Control	HG1	HG2
SR (%)	100.00 ± 0.00	100.00 ± 0.00	100.00 ± 0.00
IBW (g)	39.12 ± 0.25	39.63 ± 0.51	39.51 ± 0.09
FBW (g)	57.77 ± 0.27 ^a^	59.45 ± 0.47 ^b^	58.00 ± 0.22 ^a^
WGR (%)	47.67 ± 0.26 ^a^	50.02 ± 0.91 ^b^	46.78 ± 0.22 ^a^
SGR (%·d^−1^)	0.59 ± 0.01 ^a^	0.62 ± 0.01 ^b^	0.58 ± 0.01 ^a^
FR (%·d^−1^)	0.99 ± 0.01	0.99 ± 0.01	0.99 ± 0.02
FCR	1.45 ± 0.02 ^b^	1.38 ± 0.03 ^a^	1.47 ± 0.01 ^b^
PER	1.41 ± 0.01 ^a^	1.46 ± 0.02 ^b^	1.39 ± 0.01 ^a^
PR (%)	22.05 ± 0.22 ^a^	23.27 ± 0.25 ^b^	21.91 ± 0.12 ^a^

Note: Values in the same column with different letter superscripts indicate a significant difference (*p* < 0.05), while those with no or the same small letter superscripts mean no significant difference (*p* > 0.05). The same applies below.

**Table 2:** Effects of dietary carbohydrate levels on body shape index of Chinese perch at low water temperature (*n* = 9).

Parameters	Control	HG1	HG2
HSI (%)	1.59 ± 0.04	1.84 ± 0.12	1.83 ± 0.17
VSI (%)	7.10 ± 0.13 ^a^	7.92 ± 0.28 ^ab^	8.03 ± 0.36 ^b^
CF (g/cm^3^)	2.19 ± 0.03 ^b^	2.19 ± 0.02 ^b^	2.08 ± 0.03 ^a^

Note: Values with different letter superscripts indicate a significant difference (*p* < 0.05), while those with no or the same small letter superscripts mean no significant difference (*p* > 0.05).

**Table 3:** Effects of dietary carbohydrate levels on body composition index of Chinese perch at low water temperature (*n* = 9).

Parameters	Control	HG1	HG2
Crude lipid (%)	4.86 ± 0.14	4.62 ± 0.04	4.74 ± 0.04
Crude protein (%)	15.62 ± 0.22	15.71 ± 0.11	15.81 ± 0.11
Ash (%)	5.13 ± 0.03	5.11 ± 0.17	5.37 ± 0.08
Moisture (%)	74.84 ± 0.40	74.95 ± 0.22	74.58 ± 0.34

**Table 4:** Experimental feed formula (% dry matter).

Ingredients	Control	HG1	HG2
Fish meal ^1^	55	55	55
Casein	5	5	5
Fermented soybean meal	6	6	7
Poultry by-product meal	5	5	4
Gelatin	3	3	3
Corn starch	6	10	12
Fish oil	5	1.5	0.5
Soybean oil	5	1.5	0.5
Ca(H_2_PO_4_)_2_	2	2	2
Choline chloride	0.5	0.5	0.5
Premix ^2^	4	4	4
DMPT (C_5_H_11_SO_2_Br)	0.5	0.5	0.5
Bile acids	0.04	0.04	0.04
Carboxymethyl cellulose	1	1	1
Gum tragacanth	1	2	2
Microcrystalline cellulose	0.96	2.96	2.96
Total	100	100	100
Nutrient composition			
Crude protein (%DM)	49.73	49.11	49.24
Crude lipid (%DM)	15.79	8.93	6.91
Carbohydrate (%DM)	8.13	12.03	14.15
Ash (%DM)	12.47	12.30	12.43
Gross energy (KJ/g)	19.36	17.20	16.81

^1^ Wuhan Zhengda Aquatic Co., Ltd., imported from USA, produced in Wuhan, Hubei, dried and powdered after degreasing; ^2^ Premix in the feed of Chinese perch (per kg of feed): CaHPO_4_ 94.9 g, KCl 5.45 g, MgSO_4_ 4 g, NaCl 3.8 g, CuSO_4_ 25 mg, FeSO_4_ 407 mg, ZnSO_4_ 198 mg, MnSO_4_ 36 mg, Na_2_SeO_3_ 1.8 mg, KI 1.4 mg, Na_2_MoO_4_ 0.34 mg, CoSO_4_ 0.09 mg, KF 0.8 mg, inositol 600 mg, vitamin A 40 mg, vitamin D_3_ 0.06 mg, vitamin E 200 mg, vitamin K_3_ 10 mg, vitamin B_1_ (thiamine) 15 mg, vitamin B_2_ (riboflavin) 25 mg, vitamin B_6_ 20 mg, pantothenic acid 50 mg, vitamin B_3_ (nicotinic acid) 200 mg, biotin 3.2 mg, vitamin B_12_ 0.1 mg, folic acid 10 mg, vitamin C 210 mg.

**Table 5:** Real-time primer sequence for Chinese perch.

Primer	Primer Sequence (5′-3′)	Tm (°C)
sc-*rpl13a*-F	CACCCTATGACAAGAGGAAGC	59
sc-*rpl13a-R*	TGTGCCAGACGCCCAAG	
sc-*gs*-F	TGGATTGATGGAACTGGAGAG	58
sc-*gs*-R	CCACTCAGGCAGGTCTTC	
sc-*gdh*-F	GACGACGACCCCAACTTCT	58
sc-*gdh*-R	GACCCGCTTCCTCTTCTGC	
sc-*ampd*-F	CATTTCCTTCCCGTGTT	58
sc-*ampd*-R	TCTGTCTGCGGAGTTGGT	
sc-*rhag*-F	TGATTGGATTAGTGGCTGGCATA	58
sc-*rhag*-R	GTGGACACCGCAGGTATCTT	
sc-*rhbg*-F	AAGACGCAGCAACCAACAT	58
sc-*rhbg*-R	CCAAGGCACCGAAGAGGAT	
sc-*rhcg*-F	ACATCCAGAACTCCACTCTT	60
sc-*rhcg*-R	AGATGACACCACAGCAGAA	
sc-*gk*-F	AAGGTGGAGACCAAGAAC	58
sc-*gk*-R	TGCCCTTGTCAATGTCC	
sc-*pk*-F	CGCCCTCGCTGTCCTATTA	57
sc-*pk*-R	TGCCGAAGTTGACCCTGTTG	
sc-*pparα*-F	AGCAGAGAAGGACGTCAG	58
sc-*pparα*-R	TTCCTTCTCGGCATGCTG	
sc-*srebp1*-F	CTCCCTCCTTTCTGTCGGCTC	58
sc-*srebp1*-R	TCATTTGCTGGCAGTCGTGG	
sc-*lkb1*-F	GACGGGGCACTTAAAATC	58
sc-*lkb1*-R	GTGTTACTCCAGCAGACCAAA	
sc-*ampk*-F	GGGATGCAAACCAAGATG	54
sc-*ampk*-R	ACAGACCCAGAGCGGAGA	
sc-*eef2*-F	TCTGCTGTTATCCCGCCT	58
sc-*eef2*-R	TCGCCATCACTCCTCCTCT	
Sc-*s6k1*-F	CCTTCAAACCTTTCCTGCAATC	58
Sc-*s6k1*-R	ATTTAACTGGGCTGAGAGGTG	
sc-*mtor*-F	GCATCAACGAGAGCACCA	55
sc-*mtor*-R	CGCTTCAAAATTCATAACCG	
sc-*mafbx*-F	ACCGCATGGAGAACATCAT	58
sc-*mafbx*-R	GCAGGTCAGTCAGAGTCAT	
Sc-*murf1*-F	AGACACAGACAGACTTACGGAGAG	58
Sc-*murf1*-R	AGAGGACGCACCACCTGAC	

## Data Availability

All data are available from the corresponding author by request.

## Abbreviations

The following abbreviations are used in this manuscript:

ACC	Acetyl-CoA carboxy-lase
AKG	α-ketoglutaric acid
AMPD	Adenosine monophosphate deaminase
AMPK	AMP-activated protein kinase
CF	Condition factor
eEF2	Eukaryotic elongation factor 2
FBW	Final body weight
FCR	Feed conversion ratio
FR	Feeding rate
G6Pase	Glucose-6-phosphatase
GDH	Glutamate dehydrogenase
GK	Glucokinase
Gln	Glutamine
Glu	Glutamate
GLS	Glutaminase
GS	Glutamine synthetase
HSI	Hepatosomatic index
IBW	Initial body weight
LKB1	Liver kinase B1
MAFbx	Muscle atrophy F-box
mTOR	Mammalian target of rapamycin
MuRF1	Muscle-specific RING finger protein-1
PEPCK	Phosphoenolpyruvate carboxykinase
PER	Protein efficiency ratio
PK	Pyruvate kinase
PPARα	Peroxisome proliferators-activated receptors
PR	Protein retention rate
Rhag	Rh a glycoprotein
Rhbg	Rh b glycoprotein
Rhcg	Rh c glycoprotein
S6K1	Translational regulators S6 kinase 1
SGR	Specific growth rate
SR	Survival rate
SREBP1	Sterol regulatory element binding protein 1
VSI	Viscerosomatic index
WGR	Weight gain rate
